# Supplementary material for: Heat adaptation of phage T7 under an extended genetic code
Source: Virus Evol. 2021 Dec 1;7(2):veab100. doi: 10.1093/ve/veab100 (PMC8923235; doi:10.1093/ve/veab100)
Supplement: veab100_Supp [file veab100_supp.zip › Sequencing_Analysis.zip!/Sequencing_Analysis/S1/evidence/JC_SIDE_1_60.html]

BRESEQ :: Evidence


| New junction evidence | | | | | | | | | | | |
| --- | --- | --- | --- | --- | --- | --- | --- | --- | --- | --- | --- |
|  | | seq id | position | reads (cov) | reads (cov) | score | skew | freq | annotation | gene | product |
| \* | ? | NC\_001604.1 | = 19635 | 4193 (1.000) | 12 (0.000) | 11/590 | 44.2 | 0.29% | intergenic (–/–) | *–/–* | –/– |
|  | ? | NC\_001604.1 | 19645 = | 4138 (1.000) |  |  |  |  | intergenic (–/–) | *–/–* | –/– |
| Rejected: Coverage evenness skew score above cutoff. | | | | | | | | | | | |
| Rejected: Frequency below/above cutoff threshold. | | | | | | | | | | | |

Only 100 of 4206 total aligned reads displayed.  

`TAAGCTGTGACATTCACCACAAAGGAGACATTCTGGATGTAACGTCCGTTGTCATTAATCCTGAGGCAGACTCTAAGGGCTTACAGCGATTCCTAGCGAAACGCTTTAAGTACCTTGCGGAACTCCACGATTGCGATTGGGTGTCTCGTTGTAAGCATGAAGGCGAGACAATGCGTGTATACTTTAAGGAGGTATAAGTTATGGGTAAGAAAGTTAAGAAGGCCGTGAAGAAAGTCACCAAGTCCGTTAAGAAAGTCGTTAAGGAAGGGGCTCGTCCGGTTAAACAGGTTGCTGGCGGTCTAGCTGGTCTGGCTGGTGGTACTGGTGAAGCACAGATGGTGGAAGTACCACAAGCTGCCGCACAGATTGTTGACGTACCTGAGAAAGAGGTTTCCACTGAGGACGAAGCACAGACAGAAAGCGGACGCAAGAAAGCTCGTGCTGGCGGTAAGAAATCCTTGAGTGTAGCCCGTAGCTCCGGTGGCGGTATCAACATTTAATCAGGAGGTTATCGTGGAAGACTGCATTGAATGGACCGGAGGTGTCAACTCTAAGGGTTATGGTCGTAAGTGGGTTAATGGTAAACTTGTGACTCCACATA  >  NC_001604.1/19335‑19935`  

                                                                                                                                                                                                                                                                                                            `|`                                                                                                                                                                                                                                                                                                              
   `gCTGTGACATTCACCACAAAGGAGACATTCTGGATGTAACGTCCGTTGTCATTAATCCTGAGGCAGACTCTAAGGGCTTACAGCGATTCCTAGCGAAACGCTTTAAGTACCTTGCGGAACTCCACGATTGCGATTGGGTGTCTCGTTGTAAGCATGAAGGCGAGACAATGCGTGTATACTTTAAGGAGGTATAAGTTATGGGTAAGAAAGTTAAGAAGGCCGTGAAGAAAGTCACCAAGTCCGTTAAGAAAGTCGTTAAGGAAGGGGCTCGTCCGGTTAAACAGGTTGCTGGCGGTCTAGc                                                                                                                                                                                                                                                                                                           <  1:119763/301‑1 (MQ=255)`  
    `ttgtgACATTCACCACAAAGGAGACATTCTGGACGTAACGTCCGTTGTCATTAATCCTTAGGCAGACTCTAAGGGCTTACAACGATCCCTAGCGAAACGCTTTAAGTACCTTGAGGAACTCCACGATTGCGATTGGGTGTCTCGTTGTAAGCATGAAGGCGAGACAATGCGTGTATACTTTAAGGAGGTATAAGTTATGGGTAAGAAAGTTAAGAAGGCCGTGAAGAAAGTCACCAAGTCCGTTAAGAAAGTCGTTAAGGAAGGGGCTCGTCCGGTTAAACAGGTTGCTGGCGGTCTAGCt                                                                                                                                                                                                                                                                                                          <  1:328991/300‑1 (MQ=255)`  
     `agaatagctagcaacaAAGGTGAAATTGTGGATGTAACGTCCGTTGTAATTAATTCTGAGGCAGACTCTAAGGGCTGATAGAGATTCCTAGCGAAACGTTTTAAGTACCTTGCGGAACTCCACGATTGCGATTGGGTGTATCGTTGTAAGCATGAAGGCGAGACAATGCGTGTATACTTTAAGGAGGTATAAGTTAGGGGTAAGAAAGTTAAGAAGGCCGTGAAGAAAGTCACCAAGTCCGTTAAGAAAGTCGTTAAGGAAGGGGCTCGTCCGGTTACACAGGTTGCTGGCGGTCTAGCTg                                                                                                                                                                                                                                                                                                         <  1:389118/288‑1 (MQ=255)`  
      `atgtgtataagagacagggAGACATTCTGGATGTAACGTCCGTTGTCATTAAGCCTGAGGCAGACTCTAAGGGCTTACAGCGATTCCTAGCGAAACGCTTTAAGTACCTTGCGGAACTCCACGATTGCGATTGGGTGTCTCGTTGTAAGCATGAAGGCGAGACAATGCGTGTATACTTTAAGGAGGTATAAGTTATGGGTAAGAAAGTTAAGAAGGCCGTGAAGAAAGTCACCAAGTCCGTTAAGAAAGTCGTTAAGGAAGGGGCTCGTCCGGTTAAACAGGTTGCTGGCGGTCTAGCTgg                                                                                                                                                                                                                                                                                                        <  1:170028/284‑1 (MQ=255)`  
             `taaccacAAAGGAGACATTCTGGATGTAACGTCCGTTGTCATTAATCCTGAGGCAGACTCTAAGGGCTTACAGCGATTCCTAGCGAAACGCTTTAAGTACCTTGCGGAACTCCACGATTGCGATTGGGTGTCTCGTTGTAAGCATGAAGGCGAGACAATGCGTGTATACTTTAAGGAGGTATAAGTTATGGGTAAGAAAGTTAAGAAGGCCGTGAAGAAAGTCACCAAGTCCGTTAAGAAAGTCGTTAAGGAAGGGGCTCGTCCGGTTAAACAGGTTGCTGGCGGTCTAGCTGGTctggct                                                                                                                                                                                                                                                                                                 <  1:561527/299‑1 (MQ=255)`  
              `caccacAAAGGAGACATTCTGGATGTAACGTCCGTTGTCATTAATCCTGAGGCAGACTCTAAGGGCTTACAGCGATTCCTAGCGAAACGCTTTAAGTACCTTGCGGAACTCCACGATTGCGATTGGGTGTCTCGTTGTAAGCATGAAGGCGAGACAATGCGTGTATACTTTAAGGAGGTATAAGTTATGGGTAAGAAAGTTAAGAAGGCCGTGAAGAAAGTCACCAAGTCCGTTAAGAAAGTCGTTAAGGAAGGGGCTCGTCCGGTTAAACAGGTTGCTGGCGGTCTAGCTGGTctggctg                                                                                                                                                                                                                                                                                                >  1:205511/1‑301 (MQ=255)`  
               `aagagacaGGAGACATTCTGGATGTAACGTCCGTTGTCATTAATCCTGAGGCAGACTCTAAGGGCTTACAGCGATTCCTAGCGAAACGCTTTAAGTACCTTGCGGAACTCCACGATTGCGATTGGGTGTCTCGTTGTAAGCATGAAGGCGAGACAATGCGTGTATACTTTAAGGAGGTATAAGTTATGGGTAAGAAAGTTAAGAAGGCCGTGAAGAAAGTCACCAAGTCCGTTAAGAAAGTCGTTAAGGAAGGGGCTCGTCCGGTTAAACAGGTTGCTGGCGGTCTAGCTGGTctggctga                                                                                                                                                                                                                                                                                               <  1:223481/294‑2 (MQ=255)`  
                `ccacAAAGGAGACATTCTGGATGTAACGTCCGTTGTCATTAATCCTGAGACAGACTCTAAGGGCTTACAGCGATTCCTAGCGAAACGCTTTAAGTACCTTGCGGAACTCCACGATTGCGATTGGGTGTCTCGTTGTAAGCATGAAGGCGAGACAATGCGTGTATACTTTAAGGAGGTATAAGTTATGGGTAAGAAAGTTAAGAAGGCCGTGAAGAAAGTCACCAAGTCCGTTAAGAAAGTCGTTAAGGAAGGGGCTCGTCCGGTTAAACAGGTTGCTGGCGGTCTCGCTGGTCTGGAtggt                                                                                                                                                                                                                                                                                              >  1:52529/1‑301 (MQ=255)`  
                   `aaaaGGAGACATTCTGGATGTAACTTCCGTTGTCATTAAGCCTGAGGCAGACTCTAAGGGCTTACAGGGATTCCTAGCGAAACGCTTTAAGTACCTTGCGGAACTCCACGATTGCGATTGGGTGTCTCGTTGTAAGCATGAAGGCGAGACAATGCGTGTATACTTTAAGGAGGTATAAGTTATGGGTAAGAAAGTTAAGAAGGCCGTGAAGAAAGTCACCAAGTCCGTTAAGAAAGTCGTTAAGGAAGGGGCTCGTCCGGTTAAACAGGTTGCTGGCGGTCTAGCTGGTCTGGCtggtggc                                                                                                                                                                                                                                                                                           <  1:358672/300‑2 (MQ=255)`  
                      `gggAGACATTCTGGATGTAACGTCCGTTGTCATTAATCCTGAGGCAGACTCTAAGGGCTTACAGCGATTCCTAGCGAAACGCTTTAAGTACCTTGCGGAACTCCACGATTGCGATTGGGTGTCTCGTTGTAAGCATGAAGGCGAGACAATGCGTGTATACTTTAAGGAGGTATAAGTTATGGGTAAGAAAGTTAAGAAGGCCGTGAAGAAAGTCACCAAGTCCGTTAAGAAAGTCGTTAAGGAAGGGGCTCGTCCGGTTAAACAGGTTGCTGGCGGTCTAGCTGGTCTGGCTGGTGGTACt                                                                                                                                                                                                                                                                                        <  1:435977/300‑1 (MQ=255)`  
                       `ggAGACATTCTGGATGTAACGTCCGTTGTCATTAATCCTGAGGCAGACTCTAAGGGCTTACAGCGATTCCTAGCGAAACGCTTTAAGTACCTTGCGGAACTCCACGATTGCTATTGGGTGTCTCGTTGTAAGCATGAAGGCGAGACAATGCGTGTATACTTTAAGGAGGTATAAGTTATGGGTAAGAAAGTTAAGAAGGCCGTGAAGAAAGTCACCAAGTCCGTTAAGAAAGTCGTTAAGGAAGGGGCTCGTCCGGTTAAACAGGTTGCTGGCGGTCTAGCTGGTCTGGCTGGTGGTACTg                                                                                                                                                                                                                                                                                       >  1:494707/1‑301 (MQ=255)`  
                       `ggAGACATTCTGGATGTAACGTCCGTTGTCATTAATCCTGAGGCAGACTCTAAGGGCTTACAGCGATTCCTAGCGAAACGCTTTAAGTACCTTGCGGAACTCCACGATTGCGATTGGGTGTCTCGTTGTAAGCATGAAGGCGAGACAATGCGTGTATACTTTAAGGAGGTATAAGTTATGGTTAAGAAAGTTAAGAAGGCCGTGAAGAAAGTAACCAAGTCCGTTAAGAAAGTCGTTAAGGAAGGGGCTCGTCCGGTTAAACAGGTTGCTGGCGGTCTAGCTGGTCTGGCTGGTGGTACTg                                                                                                                                                                                                                                                                                       >  1:408650/1‑301 (MQ=255)`  
                       `ggAGACATTCTGGATGTAACGTCCGTTGTCATTAATCCTGAGGCAGACTCTAAGGGCTTACAGCGATTCCTAGCGAAACGCTTTAAGTACCTTGCGGAACTCCACGATTGCGATTGGGTGTCTCGTTGTAAGCATGAAGGCGAGACAATGCGTGTATACTTTAAGGAGGTATAAGTTATGGGTAAGAAAGTTAAGAAGGCCGTGAAGAAAGTCACCAAGTCCGTTAAGAAAGTCGTTAAGGAAGGGGCTCGTCCGGTTAAACAGGTTGCTGGCGGTCTAGCTGGTCTGGCTGGTGGTACTg                                                                                                                                                                                                                                                                                       >  1:67010/1‑301 (MQ=255)`  
                        `gagaCATTCTGGATGTAACGTCCGTTGTCATTAATCCTGAGGCAGACTCTAAGGGCTTACAGCGATTCCTAGCGAAACGCTTTAAGTACCTTGCGGAACTCCACGATTGCGATTGGGTGTCTCGTTGTAAGCATGAAGGCGAGACAATGCGTGTATACTTTGAGGAGGTATAAGTTATGGGTAAGAAAGTTAAGAAGGCCGTGAAGAAAGTCACCAAGTCCGTTAAGAAAGTCGTTAAGGAAGGGGCTCGTCCGGTTAAACAGGTTGCTGGCGGTCTAGCTGGTCTGGCTGGTGGTACTgg                                                                                                                                                                                                                                                                                      <  1:112531/301‑1 (MQ=255)`  
                        `gagaCATTCTGGATGTAACGTCCGTTGTCATTAATCCTGAGGCAGACTCTAAGGGCTTACAGCGATTCCTAGCGAAACGCTTTAAGTACCTTGCGGAACTCCACGATTGCGATTGGGTGTCTCGTTGTAAGCATGAAGGCGAGACAATGCGTGTATACTTTAAGGAGGTATAAGTTATGGGTAAGAAAGTTAAGAAGGCCGTGAAGAAAGTCACCAAGTCCGTTAAGAAAGTCGTTAAGGAAGGGGCTCGTCCGGTTAAACAGGTTGCTGGCGGTCTAGCTGGTCTGGCTGGTGGTACTgg                                                                                                                                                                                                                                                                                      <  1:156963/301‑1 (MQ=255)`  
                        `aaagagacaaggATGTAACGTCCGTTGTCATTAATCCTGAGGCAGACTCTAAGGGCTTACAGCGATTCCTAGGGAAACGCTTTAAGTACCTAGCGGAACTCCACGATTGCGATTGAGTGTATCGTTGTAAGCATGAAGGCGAGACAATGAGTGTATAATTTAAGGAGGTATAAGTTATGGGTAAGAAAGTTAAGAAGGCCGTGAAGAAAGTCACCAAGTCCGTTAAGAAAGTCGTTAAGGAAGGGGCTCGTCCGGTTAAACAGGTTGCTGGCGGTCTAGCTGGTCTGGCTGGTGGTACTgg                                                                                                                                                                                                                                                                                      <  1:657814/291‑1 (MQ=255)`  
                          `gaCATACTGGAGGTACCCTCCGTTGTCATTAATCCTGAGGCAGAGTTTAATGGCTTACATCTATTCCGAGCGAAACGCTTTAAGTACCTTGCTGAACTCCACGTGTGCGATTGGGTGTCTCCTTGTAAGCATGAAGGCGAGACAATGCGTGTATACTTTAAGGAGGTATAAGTTATGGGTAAGAAAGTTAAGAAGGCCGTGAAGAAAGTCACCAAGTCCGTTAAGAAAGTCGTTAAGGAAGGGGCTCGTCCGGTTAAACAGGTTGCTGGCGGTCTAGCTGGTCTGGCTGGTGGTACTGGTg                                                                                                                                                                                                                                                                                    <  1:283160/301‑1 (MQ=255)`  
                             `tcggagatgtgtataagagacagaaTTAATCCTGAGGCAGACTCGAAGGGCTAGCAGCGATTCCTAGCGAAACGCTGTAAGTACCTTGCGGAACTCCACGATTGTGATTGGGTGTCTCGTTGTAAGCATGAAGGCGAGACAATGCGTGTATACTTTAAGGAGGTATAAGTTATGGGTAAGAAAGTTAAGAAGGCCGTGAAGAAAGTCACCAAGTCCGTTAAGAAAGTCGTTAAGGAAGGGGCTCGTCCGGTTAAACAGGTTGCTGGCGGTCTAGCTGGTCTGGCTGGTGGTACTGGTGAAg                                                                                                                                                                                                                                                                                 <  1:461125/277‑1 (MQ=255)`  
                                `ggctcggagatgtgtataagagacagtCCTGAGGCAGACTCTAAGGGCTTACAGCGATTCCTAGCGAAACGCTTTAAGTACCTTGCGGAACTCCACGATTGCGATTGGGTGTCTCGTTGTAAGCATGAAGGCGAGACAATGCGTGTATACTTTAAGGAGGTATAAGTTATGGGTAAGAAAGTTAAGAAGTCCGTGAAGAAAGTCACCAAGTCCGTTAAGAAAGTCGTTAAGGAAGGGGCTCGTCCGGTTAAACAGGTTGCTGGCGGTCTAGCTGGTCTGGCTGGTGGTACTGGTGAAGcac                                                                                                                                                                                                                                                                              <  1:236063/275‑1 (MQ=255)`  
                                          `cGTCCGTTGTCATTAATCCTGAGGCAGACTCTAAGGGCTTACAGCGATTCCTAGCGAAACGCTTTAAGTACCTTGCGGAACTCCACGATTGCGATTGGGTGTCTCGTTTTAAGCATGAAGGCGAGACAATGCGTGTATACTTTAAGGAGGTATAAGTTATGGGTAAGAAAGTTAAGAAGGCCGTGAAGAAAGTCACCAAGTCCGTTAAGAAAGTCGTTAAGGAAGGGGCTCGTCCGGTTAAACAGGTTGCTGGCGGTCTAGCTGGTCTGGCTGGTGGTACTGGTGAAGCACAGATGGTGGa                                                                                                                                                                                                                                                                    <  1:622635/301‑1 (MQ=255)`  
                                               `gTTGTCATTAATCCTGGGGGAGAATTTAGAGGCTTACAGCGAATCCTAGCAAAACGCGTTAAGGACCTTGCGGAACTCCAGGATTGCGATTTGGTGTCTCGTTGTAAGAAAGAAGGGGAGGCAATGCGTGTATACTTTAAGGAGGTATAAGTTATGGGTAAGAAAGTTAAGAAGGCCGTGAAGAAAGTCACCAAGGCCGTTAAGAAAGTCGTTAAGGAAGGGGCTCGTCCGGGTAAACAGGTTGCTGGCGGTCTAGCTGGTCTGGCTGGTGGTACTGGTGAAGCACAGATGGTGGAAGTAc                                                                                                                                                                                                                                                               <  1:489075/301‑1 (MQ=255)`  
                                                `ttGTCATTAATCCTGAGGCAGACTCTAAGGGCTTACAGCGATTCCTAGCGAAACGCTTTAAGTACCTTGCGGAACTCCACGATTGCGATTGGGTGTCTCGTTTTAAGCATGAAGGCGAGACAATGCGTGTATACTTTAAGGAGGTATAAGTTATGGGTATGAACGTTAAGAATGCCGTTAAGAAAGTCCCCAAGTCCGTTAAGAATGTCGTTAAGGAAGGGGCTCGTCCGGTTAAACAGGTTGCTGGCGGTCTAGCTGGTCTGTCTGGTGGTACTGGTGACGCACAGATGATGGAAGTAcc                                                                                                                                                                                                                                                              >  1:396305/1‑301 (MQ=255)`  
                                                  `tgtataagagacagggCAGACTCTAAGGGCTTACAGCGATTCCTAGCGAAACGCTTTAAGTACCTTGCGGAACTCCACGATTGCGATTGGGTGTCTCGTTGTAAGCATGAAGGCGAGACAATGCGTGTATACTTTAAGGAGGTATAAGTTATGGGTAAGAAAGTTAAGAAGGCCGTGAAGAAAGTCACCAAGTCCGTTAAGAAAGTCGTTAAGGAAGGGGCTCGTCCGGTTAAACAGGTTGCTGGCGGTCTAGCTGGTCTGGCTGGTGGTACTGGTGAAGCACAGATGGTGGAAGTACcac                                                                                                                                                                                                                                                            <  1:479150/287‑1 (MQ=255)`  
                                                  `gtaaTTAATCCTGAGGCAGACTCTAAGGGCTTACAGCGATTCCTAGCGAAACGCTTTAAGTACCTTGCGGAACTCCACGATTGCGATTGGGTGTCTCGTTGTAAGCATGAAGGCGAGACAATGCGTGTATACTTTAAGGAGGTATAAGTTATGGGTAAGAAAGTTAAGAAGGCCGTGAAGAAAGTCACCAAGTCCGTTAAGAAAGTCGTTAAGGAAGGGGCTCGTCCGGTTAAACAGGTTGCTGGCGGTCTAGCTGGTCTGGCTGGTGGTACTGGTGAAGCACAGATGGTGGAAGTACcac                                                                                                                                                                                                                                                            <  1:542248/298‑1 (MQ=255)`  
                                                  `gTCATTAATCCTGAGGCAGACTCTAAGGGCTTACAGCGATTCCTAGCGAAACGCTTTAAGTACCTTGCGGAACTCCACGATTGCGATTGGGTGTCTCGTTGTAAGCATGAAGGCGAGACAATGCGTGTATACTTTAAGGAGGTATAAGTTATGGGTAAGAAAGTTAAGAAGGCCGTGAAGAAAGTCACCAAGTCCGTTAAGAAAGTCGTTAAGGAAGGGGCTCGTCCGGTTAAACAGGTTGCTGGCGGTCTAGCTGGTCTGGCTGGTGGTCCTGGTGAAGCACAGATGGTGGAAGtgtgtc                                                                                                                                                                                                                                                            >  1:83879/1‑296 (MQ=255)`  
                                                  `gTCATTAATCCTGAGGCAGACTCTAAGGGCTTACAGCGATTCCTAGCGAAACGCTTTAAGTACCTTGCGGAACTCCACGATTGCGATTGGGTGTCTCGTTGTAAGCATGAAGGCGAGACAATGCGTGTATACTTTAAGGAGGTATAAGTTATGGGTAAGAAAGTTAAGAAGGCCGTGAAGAAAGTCACCAAGTCCGTTAAGAAAGTCGTTAAGGAAGGGGCTCGTCCGGTTAAACAGGTTGCTGGCGGTCTAGCTGGTATGGCTGGTGGTACTGGTGAAGCACAGATGGTGGAAGTACcac                                                                                                                                                                                                                                                            >  1:576642/1‑301 (MQ=255)`  
                                                    `cATTAATCCTGAGGCAGACTCTAAGGGCTTACAGCGATTCCTAGCGAAACGCTTTAAGTACCTTGCGGAACTCCACGATTGCGATTGGGTGTCTCGTTGTAAGCATGAAGGCGAGACAATGCGTGTATACTTTAAGGAGGTATAAGTTATGGGTAAGAAAGTTAAGAAGGCCGTGAAGAAAGTCACCAAGTCCGTTAAGAAAGTCGTTAAGGAAGGGGCTCGTCCGGTTAAACAGGTTGCTGGCGGTCTAGCTGGTCTGGCTGGTGGGACTGGTGAAGCACAGATGGTGGAAGTACCCCaa                                                                                                                                                                                                                                                          >  1:307295/1‑301 (MQ=255)`  
                                                        `tgtataagagacagctctAAGTGCTTACAGCGATTCCTAGCGAAACGCTTTAAGTACCTTGCGGAACTCCACGATTGCGATTGGGTGTCTCGTTGTAAGCATGAAGGCGAGACAATGCGTGTATACTTTAAGGAGGTATAAGTTATGGGTAAGAAAGTTAAGAAGGCCGTGAAGAAAGTCACCAAGTCCGTTAAGAAAGTCGTTAAGGAAGGGGCTCGTCCGGTTAAACAGGTTGCTGGCGGTCTAGCTGGTCTGGCTGGTGGTACTGGTGAAGCACAGATGGTGGAAGTACCACAAGcag                                                                                                                                                                                                                                                      <  1:513543/287‑3 (MQ=255)`  
                                                          `tCCTGAGGCAGACTCTAAGGGCTTACAGCGATTCCTAGCGAAACGCTTTAAGTACCTTGCGGAACTCCACGATTGCGATTGGGTGTCTCGTTGTAAGCATGAAGGCGAGACAATGCGTGTATACTTTAAGGAGGTATAAGTTATGGGTAAGAAAGTTAAGAAGGCCGTGAAGAAAGTCACCAAGTCCGTTAAGAAAGTCGTTAAGGAAGGGGCTCGTCCGGTTAAACAGGTTGCTGGCGGTCTAGCTGGTCTGGCTGGTGGTACTGGTGCAGCACAGATGGTGGAAGTACCACAAGCTGcc                                                                                                                                                                                                                                                    >  1:247146/1‑301 (MQ=255)`  
                                                                      `ctctAAGGGCTTACAGCGATTCCTAGCGAAACGCTTTAAGTACCTTGCGGAACTCCACGATTGCGATTGGGTGTCTCGTTGTAAGCATGAAGGCGAGACAATGCGTGTATACTTTAAGGAGGTATAAGTTATGGGTAAGAAAGTTAAGAAGGCCGTGAAGAAAGTCACCAAGTCCGTTAAGAAAGTCGTTAAGGAAGGGGCTGGTTCGATTAAAAAGGTGGCGGGCGGTCTAGCTGGTCTGGCTGGTGGTACTGGTGAAGCAAAGAGGGAGGAAGAACCACAAGCGGCAGCACAGAttgtt                                                                                                                                                                                                                                        >  1:143455/1‑301 (MQ=255)`  
                                                                      `ctctAAGGGCTTACAGCGATTCCTAGCGAAACGCTTTAAGTACCTTGCGGAACTCCACGATTGCGATTGGGTGTCTCGTTGTAAGCATGAAGGCGAGACAATGCGTGTATACTTTAAGGAGGTATAAGTTATGGGTAAGAAAGTTAAGAAGGCCGTGAAGAAAGTCACCAAGTCCGTTAAGAAAGTCGTTAAGGAAGGGGCTCGTCCGGTTAAACAGGTTGCTGGCGGTCTAGCTGGTCTGGCTGGTGGTACTGGTGAAGCACAGAtgctgtctcttatacacatctccgagcccacgaga                                                                                                                                                                                                                                        >  1:568547/1‑271 (MQ=255)`  
                                                                      `atctAAGGGCTTACAGCGATTCCTAGCGAAACGCTTTAAGTAACTTGCGGAACTCCACGATTGCGATTGGGTGTCTCGTTGTAAGCATGAAGGAGAGACAATGCGTGTATAATAAAAGGAGGTATAAGTAATGGGTAAGAAAGTTAAGAAGGACGTGAAGAAAGTCACCAAGTCAGTTAAGAAAGGCGTTAAGGAAGGGGATCGTCCGGTTAAACAGGTTGCTGGCGGTCTAGCTGGTCTGGCTGGTGGTACTGGTGAAGCAGAGATGGTGGAAGTACCATAAGATGCCGCACAGAttgtt                                                                                                                                                                                                                                        >  1:519599/2‑301 (MQ=255)`  
                                                                        `ctAAGGGCTTACAGCGATTCCTAGCGAAACGCTTTAAGTACCTTGCGGAACTCCACGATGGCGATGGGGTGTCTCGTTTTAAGCATGAAGGCGAGACAATGCGTGTATACTTTAAGGAGGTATAAGTTATGGGTAAGAAAGTTAAGAAGGCCGTGAAGAAAGTCACCAAGTCCGTTAAGAAAGTCGTTAAGGAAGGGGCTCGTCCGGTTAAACAGGTTGCTGGCGGTCTAGCTGGTCTGGCTGGTGGTACTGGTGAAGCACAGATGGTGGAAGTACCACAAGCTGCCGCACAGATTGTTGa                                                                                                                                                                                                                                      <  1:19877/301‑1 (MQ=255)`  
                                                                          `caGGGCTTACAGCGATGCCTAGCGAAACGCTTTAAGTACCTTGCGGAACTCCACGATTGCGATTGGGTGTCTCGTTGTAAGCATGAAGGCGAGACAATGCGTGTATACTTTAAGGAGGTATAAGTTATGGGTAAGAAAGTTAAGAAGGCCGTGAAGAAAGTCACCAAGTCCGTTAAGAAAGTCGTTAAGGAAGGGGCTCGTCCGGTTAAACAGGTTGCTGGCGGTCTAGCTGGTCTGGCTGGTGGTACTGGTGAAGCACAGATGGTGGAAGTACCACAAGCTGCCGCACAGATTGTTGACg                                                                                                                                                                                                                                    <  1:163395/300‑1 (MQ=255)`  
                                                                          `aaGGGCTTACAGCGATACCTACCGAAAGGTTTTAAGTACCTTGCGGAACTCCACTATTGCGATTGGGTGGCTCGTTGTAAGCATTAAGGGCAGACAATCCGTGTATAATTTAAGGAGGTATAAGTGATGGGTAAGAAAGTTAAGAAGGCCGTGACGAAAGTTACCAAGTACGTTAAGAAAGTCGTTAAGGAAGGGGCTCGTCCGGTTAAACAGGTTGCTGGCGGTCTAGCTGGTCTGGCTGGTGGTACTGGTGAAGCACAGATGGTGGAAGTACCACAAGCTGCCGCACAGATTGTTGACg                                                                                                                                                                                                                                    <  1:213632/301‑1 (MQ=255)`  
                                                                             `ggCTTACAGCGATTCCTAGCGAAACGCTTTAAGTACCTTGCGGAACTCCACGATTGCGATTGGGTGTCTCGTTGTAAGCATGAAGGCGAGACAATGCGTGTATACTTTAAGGAGGTATAAGTTATGGGTAAGAAAGTTAAGAAGGCCGTGAAGAAAGTCACCAAGTCCGTTAAGAAAGTCGTTAAGGAAGGGGCTCGTCCGGTTAAACAGGTTGCTGGCGGTCTCGCTGGTCTGGCTGGTGGTACTGGTGAAGCACAGATGGTGGAAGTACCACAAGCTGCCGCACAGATTGTTGACgctg                                                                                                                                                                                                                                 >  1:634218/1‑298 (MQ=255)`  
                                                                                         `ttCCTAGCGAAACGCTTTAAGTACCTTGCGGAACTCCACGATTGCGATTGGGTGTCTCGTTGTAAGCATGAAGGCGAGACAATGCGTGTATACTTTAAGGAGGTATAAGTTATGGGTAAGAAAGTTAAGAAGGCCGTGAAGAAAGTCACCAAGTCCGTTAAGAAAGTCGTTAAGGAAGGGGCTCGTCCGGTTAAACAGGTTGCTGGCGGTCTAGCTGGTCTGGCTGGTGGTACTGGTGAAGCACAGATGGTGGAAGTACCACAAGCTGCCGCACAGATTGTTGACGTACCTGAGAAAGAgg                                                                                                                                                                                                                     >  1:260734/1‑301 (MQ=255)`  
                                                                                         `ttCCGAGCGAAACGCTTTAAGTACCTGGCGGAACGCAAAGGTTGCGATTGGGTGGCTCGTTGTAAGAATGAAGGCGAGACAATGCGTGTATACTGTAAGGAGGTATAAGTTATGGGTAAGAAAGTGAAGAAGGCCGTGAAGAAAGTCACCAAGTCCGTTAAGAAAGGCGTGAAGGAAGGGGCTAGTCCGGTTAAACAGGTTGCTGGCGGTCTAGCTGGTCTGGCTGGTGGTACTGGTGAAGCACAGAGGGTGGAAGGACCACAAGCTGCAGCACAGATTGTTGAAGTACCTGAGAAAGAgg                                                                                                                                                                                                                     <  1:429672/301‑1 (MQ=255)`  
                                                                                                `cGAAACGCTTTAAGTACCTTGCGGAACTCCACGATTGCGATTGGGTGTCTCGTTGTAAGCATGAAGGCGAGACAATGCGTGTATACTTGAAGGAGGTATAAGTTATGGGTAAGAAAGTTAAGAAGGCCGTGAAGAAAGTCACCAAGTCCGTTAAGAAAGTCGTTAAGGAAGGGGCTCGTCCGGTTAAACAGGTTGCTGGCGGTCTAGCTGGTCTGGCTGGTGGTACTGGTGAAGCACAGATGGTGGAAGTACCACAAGCTTCCGCACAGATTGTTGACGTACCTGAGAAAGAGGTTTCCAc                                                                                                                                                                                                              <  1:18157/301‑1 (MQ=255)`  
                                                                                                       `cTTTAAGTACCTTGCGGAACTCCAAGATTGCGATTGGGGGTCTAGGTGTAAGCATGAAGGCGAGACAATGCGTGTATACTTTAAGGAGGTATAAGTTATGGGTAAGAAAGTTAAGAAGGCCGTGAAGAAAGTCACCAAGTCCGTTAAGAAAGACGTTAAGGAAGGGGCTCGTCCGGTTAAACAGGTTGCTGGCGGTCTAGCTGGTCTGGCTGGTGGTACTGGTGAAGCACAGATGGTGGAAGTACAACAAGCTGCCGCAAAGATTGTTGACGTACCTGAGAAAGAGGTTTACAGTGAGGaa                                                                                                                                                                                                       >  1:94071/1‑300 (MQ=255)`  
                                                                                                               `aCCTTGCGGAACTCCACGATTGCGATTGGGTGTCTCGTTGTAAGCATGAAGGCGAGACAATGCGTGTATACTTTAAGGAGGTATAAGTTATGGGTAAGAAAGTTAAGAAGGCCGTGAAGAAAGTCACCAAGTCCGTTAAGAAAGTCGTTAAGGAAGGGGCTCGTCCGGTTAAACAGGTTGCTGGCGGTCTAGCTGGTCTGGCTGGTGGTACTGGTGAAGCACAGATGGTGGAAGTACCACAAGCTGCCGCACAGATTGTTTACGTACCTGAGAAAGAGGTTTCCACTGAGGACGAAGcaca                                                                                                                                                                                               >  1:242191/1‑301 (MQ=255)`  
                                                                                                                  `ttGCGGAACTCCACGATTTGGATTGGGTGTCTCGTTGTAAGCATGAAGGCGAGACAATGCGTGTATACTTTAAGAAGGTATAAGTTATGGGTAAGAAAGTTAAGAAGGCCGTGAAGAAAGTGACCAAGTCCGTTAAGAAAGTCGTTAAGGAGGGGGCTCGTCCGGTTAAACAGGTTGCTGGCGGTCGAGCTGGTCTGGCTGGTGGTACTGGTGAAGCACAGATGGTGGAAGTACCACAAGCTGCCGCACAGATTGTTGACGTACCTGAGAAAGAGGTTTCCACTGAGGACGAAGCacagac                                                                                                                                                                                            <  1:501861/301‑1 (MQ=255)`  
                                                                                                                   `aagagacagccACGATTGCGATTGGGTGTCTCGTTGTAAGCATGAAGGCGAGACAATGCGTGTATACTTTAAGGAGGTATAAGTTATGGGTAAGAAAGTTAAGAAGGCCGTGAAGAAAGTCACCAAGTCCGTTAAGAAAGTCGTTAAGGAAGGGGCTCGTCCGGTTAAACAGGTTGCTGGCGGTCTAGCTGGTCTGGCTGGTGGTACTGGTGAAGCACAGATGGTGGAAGTACCACAAGCTGCCGCACAGATTGTTGACGTACCTGAGAAAGAGGTTTCCACTGAGGACGAAGCacagaca                                                                                                                                                                                           <  1:192027/292‑1 (MQ=255)`  
                                                                                                                    `gCGGAACTCCACGATTGCGATTGGGTGTCTCGTTGTAAGCATGAAGGCGAGACAATGCGTGTATACTTTAAGGAGGTATAAGTTATGGGTAAGAAAGTTAAGAAGGCCGTGAAGAAAGTCACCAAGTCCGTTAAGAAAGTCGTTAAGGAAGGGGCTCGTCCGGTTAAACAGGTTGCTGGCGGTCTAGCTGGTCTGGCTGGTGGTACTGGTGAAGCACAGATGGTGGAAGTACCACAAGCTGCCGCACAGATTGTTGACGTACATGAGCAAGAGGTTTCCACTGAGGAAGAAGAacagacag                                                                                                                                                                                          >  1:319295/1‑301 (MQ=255)`  
                                                                                                                      `ggAACGCCACGATTGGAATGGGGTGTCTTGTTGTAAGAATGAAGGCGAGAAAATGGGTGGATACTTTAAGGAGGTAGAAGTTATGGGTAAGAAAGTTAAGAAGGCCGTGAAGAAAGTCACCAAGTCCGTTGAGAAAGTCGTTAAGGAAGGGGCTCGTCCGGTTAAACAGGTGGCTGGCGGTCTAGCTGGTCTGGCTGGTGGTACTGGTGAAGCACAGATGGTGGAAGTACCACAAGCTGCCGCACAGATTGTTGACGTACCTGAGAAAGAGGTTTCCACTGAGGACGAAGCACAGACAGaa                                                                                                                                                                                        <  1:548366/301‑1 (MQ=255)`  
                                                                                                                          `cTCCACGATTGCGATTGGGTGTCTCGTTGTAAGCATGAAGGCGAGACAATGCGTGTATACTTTAAGGAGGTATAAGTTATGGGTAAGAAAGTTAAGAAGGCCGTGAAGAAAGTCACCAAGTCCGTTAAGAAAGTCGTTAAGGAAGGGGCTCGTCCGGTTAAACAGGTTGCTGGCGGTCTAGCTGGTCTGGCTGGTGGTACTGGTGAAGCACAGATGGTGGAAGTACCACAAGCTGCCGCACAGATTGTTGACGTACCTGAGAAAGAGGTTTCCACTGAGGACGAAGcacctgtctcttata                                                                                                                                                                                    >  1:138142/1‑289 (MQ=255)`  
                                                                                                                                 `attgcgattgGGTGTCTCGTTGTAAGCATGAAGGCGAGACAATGCGTGTATACTTTAAGGAGGTATAAGTTATGGGTAAGAAAGTTAAGAAGGCCGTGAAGAAAGTCACCAAGTCCGTTAAGAAAGTCGTTAAGGAAGGGGCTCGTCCGGTTAAACAGGTTGCTGGCGGTCTAGCTGGTCTGGCTGGTGGTACTGGTGAAGCACAGATGGTGGAAGTGCCACAAGCTGCCGCACAGATTGTTGACGTACCTGAGAAAGAGGTTTCCACTGAGGACGAAGCACAGACAGAAAGCGGACGCaa                                                                                                                                                                             >  1:265904/1‑301 (MQ=255)`  
                                                                                                                                    `gcgattgGGTGTCTCGTTGTAAGCATGAAGGCGAGACAATGCGTGTATACTTTAAGGAGGTATAAGTTATGGGTAAGAAAGTTAAGAAGGCCGTGAAGAAAGTCACCAAGTCCGTTAAGAAAGTCGTTAAGGAAGGGGCTCGTCCGGTTAAACAGGTTGCTGGCGGTCTAGCTGGTCTGGCTGGTGGTACTGGTGAAGCACAGATGGTGGAAGTACCACAAGCTGCCGCACAGATTGTTGACGTACCTGAGAAAGAGGTTTCCACTGAGGACGAAGCACAGACAGAAAGCGGACGCAAGaa                                                                                                                                                                          >  1:402306/1‑301 (MQ=255)`  
                                                                                                                                           `ggTGTCTCGTTGTAAGCATGAAGGCGAGACAATGCGTGTATACTTTAAGGAGGTATAAGTTATGGGTAAGAAAGTTAAGAAGGCCGTGAAGAAAGTCACCAAGTCCGTTAAGAAAGTCGTTAAGGAAGGGGCTCGTCCGGTTAAACAGGTTGCTGGCGGTCTAGCTGGTCTGGCTGGTGGTACTGGTGAAGCACAGATGGTGGAAGTACCACAAGCTGCCGCACAGATTGTTGACGTACCTGAGAAAGAGGTTTCCACTGAGGACGAAGCACAGACAGAAAGCGGACGCAAGaactgtctc                                                                                                                                                                   >  1:254567/1‑294 (MQ=255)`  
                                                                                                                                                  `cGTTGTAAGCATGAAGGCGAGACAATGCGTGTATACTTTAAGGAGGTATAAGTTATGGGTAAGAAAGTTAAGAAGGCCGTGAAGAAAGTCACCAAGTCCGTTAAGAAAGTCGTTAAGGAAGGGGCTCGTCCGGTTAAACAGGTTGCTGGCGGTCTAGCTGGTCTGGCTGGTGGTACTGGTGAAGCACAGATGGTGGAAGTACCACAAGCTGCCGCACAGATTGTTGACGTACCTGAGAAAGAGGTTTCCACTGAGGACGAAGCACAGACAGAAAGCGGACGCAAGAAAGCTCGTGCTGGCg                                                                                                                                                            >  1:78071/1‑301 (MQ=255)`  
                                                                                                                                                  `cGTTGTAAGCATGAAGGCGAGACAATGCGTGTATACTTTAAGGAGGTATAAGTTATGGGTAAGAAAGTTAAGAAGGCCGTGAAGAAAGTCACCAAGTCCGTTAAGAAAGTCGTTAAGGAAGGGGCTCGTCCGGTTAAACAGGTTGCTGGCGGTCTAGCTGGTCTGGCTGGTGGTACTGGTGAAGCACAGATGGTGGAAGTACCACAAGCTGCCGCACAGATTGTTGACGTACCTGAGAAAGAGGTTTACACGGAGGACGAAGCACAGACAGAAAGCGGACGCAAGAAAGCTCGTGCTGGCg                                                                                                                                                            >  1:553237/1‑301 (MQ=255)`  
                                                                                                                                                      `gTAAGCATGAAGGCGAGACAATGCGTGTATACTTTAAGGAGGTATAAGTTATGGGTAAGAAAGTTAAGAAGGCCGTGAAGAAAGTCACCAAGTCCGTTAAGAAAGTCGTTAAGGAAGGGGCTCGTCCGGTTAAACAGGTTGCTGGCGGTCTAGCTGGTCTGGCTGGTGGTACTGGTGAAGCACAGATGGTGGAAGTACCACAAGCTGCCGCACAGATTGTTGACGTACCTGAGAAAGAGGTTTCCACTGAGGACGAAGCACAGACAGAAAGCGGACGCAAGAAAGCTCGTGCTGGCGGTaa                                                                                                                                                        >  1:65262/1‑301 (MQ=255)`  
                                                                                                                                                            `acagaGGCGAGACAATGCGGGTATACTTTAAGGAGGTATAAGTTATGGGTAAGAAAGTTAAGAAGGACGTGAAGAAAGTCACCAAGTCCGTTAAGAAAGTCGTTAAGGAAGGGGCTCGTCCGGTTAAACAGGTTGCTGGCGGTCTAGCTGGTCTGGCTGGTGGTACTGGTGAAGCACAGATGGTGGAAGTACCACAAGCTGCCGCACAGATTGTTGACGTACCTGAGAAAGAGGTTTCCACTGAGGACGAAGCACAGACAGAAAGCGGACGCAAGAAAGCTCGTGCTGGCGGTAAGAAATc                                                                                                                                                  <  1:589816/297‑1 (MQ=255)`  
                                                                                                                                                                `aGGCGATACAATTCGTGAATACGTTAAGGAGGGATAAGTGATGGGTAAGAAAGTTAAGAAGGCCGTGAAGAAAGTCCCCCAGTCCGTTAAAAAAGTCGTTAAGGAAGGGGCTCGTCCGGTTAAACAGGTTGCTGGCGGTCTAGCTGGTCTGGCTGGTGGTACTGGTGAAGCACAGATGGTGGAAGTACCACAAGCTGCCGCACAGATTGTTGACGTACCTGAGAAAGAGGTTTCCACTGAGGACGAAGCACAGACAGAAAGCGGACGCAAGAACGCTCGTGCTGGCGGTAAGAAATCCTTg                                                                                                                                              <  1:628572/301‑1 (MQ=255)`  
                                                                                                                                                                         `tataagagacagcTTTAAGGAGGTATAAGTTATGGGTAAGAAAGTTAAGAAGGCCGTGAAGAAAGTCACCAAGTCCGTTAAGAAAGTCGTTAAGGAAGGGGCTCGTCCGGTTAAACAGGTTGCTGGCGGTCTAGCTGGTCTGGCTGGTGGTACTGGTGAAGCACAGATGGTGGAAGTACCACAAGCTGCCGCACAGATTGTTGACGTACCTGAGAAAGAGGTTTCCACTGAGGACGAAGCACAGACAGAAAGCGGACGCAAGAAAGCTAGTGCTGGCGGTAAGAAATCCTTGAGTGTAGcc                                                                                                                                     <  1:530770/289‑1 (MQ=255)`  
                                                                                                                                                                            `gCGTGTATACTTTAAGGAGGTATAAGTTATGGGTAAGAAAGTTAAGAAGGCCGTGAAGAAAGTCACCAAGTCCGTTAAGAAAGTCGTTAAGGAAGGGGCTCGTCCGGTTAAACAGGTTGCTGGCGGTCTAGCTGGTCTGGCTGGTGGTACTTGTGAAGCACAGATGGTGGAAGTACCACAAGCTGCCGCACAGATTGTTGACGTACCTGAGAAAGAGGTTTTCAATGAGGACGGAGCACAGACAGAAAGCGGACACAAGAAAACTCGTGCTGGCGGTAAGAAATCCTTGAATGTAGCCCGt                                                                                                                                  >  1:419144/1‑301 (MQ=255)`  
                                                                                                                                                                                 `tataCTTTAAGGAGGTATAAGTTATGGGTAAGAAAGTTAAGAAGGCCGTGAAGAAAGTCACCAAGTCCGTTAAGAAAGTCGTTAAGGAAGGGGCTCGTCCGGTTAAACAGGTTGCTGGCGGTCTAGCTGGTCTGGCTGGTGGTACTGGTGAAGCACAGATGGTGGAAGTACCACAAGCTGCCGCACAGATTGTTGACGTACCTGAGAAAGAGGTTTCCACTGAGGACGAAGCACAGACAGAAAGCGGACGCAAGAAAGCTCGTGCTGGCGGTAAGAAATCCTTGAGTGTAGCCCGTAGCTc                                                                                                                             <  1:71307/301‑1 (MQ=255)`  
                                                                                                                                                                                     `cTTTAAGGAGGTATAAGTTATGGGTAAGAAAGTTAAGAAGGCCGTGAAGAAAGTCACCAAGTCCGTTAAGAAAGTCGTTAAGGAAGGGGCTCGTCCGGTTAAACAGGTTGCTGGCGGTCTAGCTGGTCTGGCTGGTGGTACTGGTGAAGCACAGATGGTGGAAGTACCCCAAGCTGCCGCACAGTTTGTTGACGTACCTGAGAAAGAGGTTTCCACTGAGGACGAAGAACAGACAGAAAGCGGAAGCAAGAAAGCTCGTGCTGGCGGTAGGAAATCCTTGAGTGTAGCCGGTAGCTCCggg                                                                                                                         >  1:335874/1‑300 (MQ=255)`  
                                                                                                                                                                                       `agatgtgtataagagacaggggTAAGAAAGTTAAGAAGGCCGTGAAGAAAGTCACCAAGTCCGTTAAGAAAGTCGTTAAGGAAGGGGCTCGTCCGGTTAAACAGGTTGCTGGCGGTCTAGCTGGTCTGGCTGGTGGTACTGGTGAAGCACAGATGGTGGAAGTACCACAAGCTGCCGCACAGATTGTTGACGTACCTGAGAAAGAGGTTTCCACTGAGGACGAAGCACAGACAGAAAGCGGACGCAAGAAAGCTCGTGCTGGCGGTAAGAAATCCTTGAGTGTAGCCCGTAGCTCCGGTgg                                                                                                                       <  1:466874/282‑1 (MQ=255)`  
                                                                                                                                                                                             `aggTATAAGTTATGGGTAAGAAAGTTAAGAAGGCCGTGAAGAAAGTCACCACGTCCGTTAAGAAAGTCGTTAAGGAAGGGGCTCGTCCGGTTAAACAGGTTGCTGGCGGTCTAGCTGGTCTGGCTGGTGGTACTGGTGAAGCACAGATGGTGGAAGTACCACAAGCTGCCGCACAGATTGTTGACGTACCTGAGAAAGAGGTTTCCACTGAGGACGAAGCACAGACAGAAAGCGGACGCAAGAAAGCTCGTGCTGGCGGTAAGAAATCCTTGAGTGTATCCCGTAGCTCCGGTGGCGGGAt                                                                                                                 >  1:218553/1‑301 (MQ=255)`  
                                                                                                                                                                                              `ggTATAAGTTATGGGTAAGAAAGTTAAGAAGGCCGTGAAGAAAGTCACCAAGTCCGTTAAGAAAGTCGTTAAGGAAGGGGCTCGTCCGGTTAAACAGGTTGCTGGCGGTCTAGCTGGTCTGGCTGGTGGTACTGGTGAAGCACAGATGGTGGAAGTACCACAAGCTGCCGCTCAGATTGTTGACGTACCTGAGAAAGAGGTTTCCACTGAGGACGAAGCACAGACAGAAAGCGGACGCAAGAAAGCTCGTGCTGGCGGTAAGAAATCCTTGAGTGTAGCCCGTAGCTCCGGTGGCGGTATc                                                                                                                >  1:132183/1‑301 (MQ=255)`  
                                                                                                                                                                                              `ggTATAAGTTATGGGTAAGAAAGTTAAGAAGGCCGTGAAGAAAGTCACCAAGTCCGTTAAGAAAGTCGTTAAGGAAGGGGCTCGTCCGGTTAAACAGGTTGCTGGCGGTCTAGCTGGTCTGGCTGGTGGTACTGGTGAAGCACAGATGGTGGAAGTACCACAAGCTGCCGCACAGATTGTTGACGTACCCGAGAAAGAGGTTTCCACTGAGGACGAAGCACAGACAGAAAGCGGACGCAAGAAAGCTCGTGCTGGCGGTAAGAAATCCTTGAGTGTAGCCCGTAGCTCCGGTGGCGGTATc                                                                                                                >  1:609035/1‑301 (MQ=255)`  
                                                                                                                                                                                              `ggTATAAGTTATGGGTAAGAAAGTTAAGAAGGACGTGAAGAAAGTCACCAAGTCCGTTAAGAAAGTCGTTAAGGAAGGGGCTCGTCCGGTTAAACAGGTTGCTGGCGGTCTAGCTGGTCTGGCTGGTGGTACTGGTGAAGCACAGATGGTGGAAGTACCACAAGCTGCCGCACAGATTGTTGACGTACCTGAGAAAGAGGTTTCCACTGAGGACGAAGCACACACAGAAAGCGAACGCAAGAAAGCTCGTGCTGGCGGTAAGAAATCCTTGAGTGTAGCCCGTAGTGCCGGTGGCGGTATc                                                                                                                >  1:414373/1‑301 (MQ=255)`  
                                                                                                                                                                                                    `aGTTATTGGTAAGAAAGTTAAGAAGGCCGTGAAGAAAGTCACCAAGTCCGTTAAGAAAGTCGTTAAGGAAGGGGCTCGTCCGGTTAAACAGGTTGCTGGCGGTCTAGCTGGTCTGGCTGGTGGTACTGGTGAAGCACAGATGGTGGAAGTACCACAAGCTGCCGCACAGATTGTTGACGTACCTGAGAAAGAGGTTTCCACTGAGGACGAAGCACAGACAGAAAGCGGACGCAAGAAAGCTCGTGCTGGCGGTAAGAAATCCTTGAGTGTAGCCCGTAGCTCCGGTGGCGGTATCAACAtt                                                                                                          >  1:100073/1‑301 (MQ=255)`  
                                                                                                                                                                                                    `aGTTATGGGTAAGAAAGTTAAGAAGGCCGTGAAGAAAGTCACCAAGTCCGTTAAGAAAGTCGTTAAGGAAGGGGCTCGTCCGGTTAAACAGGTTGCTGGCGGTCTAGCTGGTCTGGCTGGTGGTACTGGTGAAGCACAGATGGTGGAAGTACCACAAGCTGCCGCACAGATTGTTGACGTACCTGAGAAAGAGGTTTCCACTGAGGACGAAGCACAGACAGAAAGCGGACGCAAGAAAGCTCGTGCTGGCGGTAAGAAATCCTTGAGTGTAGCCCGTAGCTCCGGTGGCGGTATCAACAtt                                                                                                          >  1:536972/1‑301 (MQ=255)`  
                                                                                                                                                                                                         `tGGGTAAGAAAGTTAAGAAGGCCGTGAAGAAAGTCACCAAGTCCGTTAAGAAAGTCGTTAAGGAAGGGGCTCGTCCGGTTAAACAGGTTGCTGGCGGTCTAGCTGGTCTGGCTGGTGGTACTGGTGAAGCACAGATGGTGGAAGTACCACAAGCTGCCGCACAGATTGTTGACGTACCTGAGAAAGAGGTTTCCACTGAGGACGAAGCACAGACAGAAAGCGGACGCAAGAAAGCTCGTGTTGGCGGTAAGAAATCCTTGAGTGTAGCCCGTAGCTCCGGTGGCGGTATCAACATTTAATc                                                                                                     <  1:383203/301‑1 (MQ=255)`  
                                                                                                                                                                                                         `aggggAAGAAAGTTAAGAAGGCCGTGAAGAAAGTCACCAAGTCCGTTAAGAAAGTCGTTAAGGAAGGGGCTCGTCCGGTTAAACAGGTTGCTGGCGGTCTAGCTGGTCTGGCTGGTGGTACTGGTGAAGCACAGATGGTGGAAGTACCACAAGCTGCCGCACAGATTGTTGACGTACCTGAGAAAGAGGTTTCCACTGAGGACGAAGCACAGACAGAAAGCGGACGCAAGAAAGCTCGTGCTGGCGGTAAGAAATCCTTGAGTGTAGCCCGTAGCTCCGGTGGCGGTATCAACATTTAATc                                                                                                     <  1:186421/300‑1 (MQ=255)`  
                                                                                                                                                                                                         `agggTAAGAAAATTAAGAAGGCCGGGAAGAAAGTCACCAAGTCCGTTAAGAAAGTCGTTAAGGAAGGGGCTCGTCCGGTTAAACAGGTTGCTGGCGGTCTAGCTGGTCTGGCTGGTGGTACTGGTGAAGCACAGATGGTGGAAGTACCACAAGCTGCCGCACAGATTGTTGACGTACCTGAGAAAGAGGTTTCCACTGAGGACGAAGCACAGACAGAAAGCGGACGCAAGAAAGCTCGTGCTGGCGGTAAGAAATCCTTGAGTGTAGCCCGTAGCTCCGGTGGCGGTATCAACATTTAATc                                                                                                     <  1:596216/300‑1 (MQ=255)`  
                                                                                                                                                                                                         `acaggaaGAAAGTTAAGAAGGCCGTGAAGAAAGTCACCAAGTCCGTTAAGAAAGTCGTTAAGGAAGGGGCTCGTCCGGTTAAACAGGTTGCTGGCGGTCTAGCTGGTCTGGCTGGTGGTACTGGTGAAGCACAGATGGTGGAAGTACCACAAGCTGCCGCACAGATTGTTGACGTACCTGAGAAAGAGGTTTCCACTGAGGACGAAGCACAGACAGAAAGCGGACGCAAGAAAGCTCGTGCTGGCGGTAAGAAATCCTTGAGTGTAGCCCGTAGCTCCGGTGGCGGTATCAACATTTAATc                                                                                                     <  1:603160/296‑1 (MQ=255)`  
                                                                                                                                                                                                          `gggTAAGAAAGTTAAGAAGGCCGTGAAGAAAGTGACCAAGTCCGTTAAGAAAGTCGTTAAGGAAGGGGCTCGTCCGGGTAAACAGGTTGCTGGCGGTCTAGCTGGTCTGGCTGGTGGTACTGGTGAAGCACAGATGGTGGAAGTACCACAAGCTGACGCACAGATTGTTGACGTACCTGAGAAAGAGGTTTCCAATGAGGACGAAGTACAGACAGAAAGCGGACGCAAGAAAGCCCGTGCTGGAGGTAAGAAATCCTTGAGTGTAGCCAGAAGCCCCGGTGGCGGTATCAACATTTAAACa                                                                                                    >  1:148780/1‑301 (MQ=255)`  
                                                                                                                                                                                                          `gggTAAGAAAGTTAAGAAGGCCGTGAAGAAAGTCACCAAGTCCGTTAAGAAAGTCGTTAAGGAAGGGGCTCGTCCGGTGAAACAGGTTGCTGGCGGGCTAGCTGGTCTGGCTGGAGGTACTGGTGAAGCACAGATGGTGGAAGTACCACAAGCTGCCGCACAGATTGTTGACGTAACTGAGAAAGAGGTTTCCACTGAGGACGAAGCACAGACAGAAAGCGGACGCAAGAAAGCTCGTGCTGGCGGGAAGAAAAACTTGAGTGTAGCCGGTAgaccagggggaggcagcaacagtgcatca                                                                                                    >  1:378137/1‑273 (MQ=255)`  
                                                                                                                                                                                                            `gTAAGAAAGTTAAGAAGGCCGTGAAGAAAGTCACCAAGTCCGTTAAGAAAGTAGTTAAGGAAGGGGCTCGTCCGGTTAAACAGGTTGCTGGAGGTCTAGCTGGTCTGGCTGGTGGTACTGGTGAAGCACAGATGGTGGCAGTACCACAAGCTGCCGAACCGATTGTTGACGTACCTGAGCAAGAGGTTTCCACTGAGGACGAAGCAAAGACAGAAAGCGGCAGCAAGAAAGCTCGAGCTGGCGGCACGAAGTCATTGAGTGTAGCCCGTAGCTCCGGTGGCGGTATCAACATTTAGGCagg                                                                                                  >  1:106700/1‑301 (MQ=255)`  
                                                                                                                                                                                                                `gAAAGTTACGAAGGCCGTGAAGAAAGTCACCAAGTCCGTTAAGAAAGTCGTTAAGGAAGGGGCTCGTCCGGTTAAACAGGTTGCTGGCGGTCTAGCTGGTCTGGCTGGTGGTACTGGTGAAGCACAGATGGTGGAAGTACCACAAGCTGCCGCACCGATTGTTGACGTACCTGAGAAAGAGGTTTCCACTGAGGACGAAGCACAGACAGAAAGCGGACGCAAGAAAGATCGTGCTGGCGGTAAGAAATCCTTGAGTGTAGCCCGTAGCTCCGGTGGCGGTATCAACATTTAATCAGGAGGt                                                                                              >  1:582319/1‑301 (MQ=255)`  
                                                                                                                                                                                                                 `aaaGTTAAGAAGGCCGTGAAGAAAGTCACCAAGTCCGTTAAGAAAGTCGTTAAGGAAGGGGCTCGTCCTGTTAAACAGGTTGCTGGCGGTCTAGCTGGTCTGGCTGGTGGTACTGGTGAAGCACAGATTGTGGAAGTACCACAAGCTGCCGCACAGATTGTTGACGTACCTGAGAAAGAGGTTTCCACTGAGGACGAAGCACAGACAGAAAGCGGACGCAAGAAAGGTCGTGCTGGCGGTAAGAAATCCTTGAGTGTAGCCCGTAGCTCCGGTGGCGGTATCAACATTTAATCAGGAGGtt                                                                                             <  1:454563/301‑1 (MQ=255)`  
                                                                                                                                                                                                                  `aaGTTAAGAAGGCCGTGAAGAAAGTCACCAAGTCCGTTAAGAAAGTCGTTAAGGAAGGGGCTCGTCCGGTTAAACAGGTTGCTTGCGGTCTAGCTGGTCTGGCTGGTGGTACTGGTGAAGCACAGATGGTGGAAGTACCACAAGCTGCCGCACAGATTGTTGACGTACCTGAGAAAGAGGTTTCCACTGAGGACGAAGCACAGACAGAAAGCGGACGCAAGAAAGCTCGTGCTGGCGGTAAGAAATCCTTGAGTGTAGCCCGTAGCTCCTGTGGCGGTCTCAACATTTAATCagttgtctc                                                                                            >  1:271365/1‑294 (MQ=255)`  
                                                                                                                                                                                                                     `ttAAGAAGGCGGTGAAGAAGGTCACGAAGTCCGTGAAGAAAGTAGTTAAGGAAGGGGCTCGTCCGGTTAAGCAGGTTGCTGGCGGTCTAGCTGGTCTGTCTGGGGGTACTGGTGAAGCACAGAGGGTGGAAGTACCAAAAGCTGCCGGACAGATTGTTGACGTACCTGAGAAAGAGGTTTCCACTGAGGACGAAGAACAGACAGAAAGCGGACGCAAGAAAGCTCGTGCTGGCGGTAAGAAAGACTTGAGTGTAGCACGTAGCTCCGGTGGCGGTATCAACAATTAATCAGGAGGTTATCg                                                                                         <  1:302635/301‑1 (MQ=255)`  
                                                                                                                                                                                                                     `gtaggatggCCGTTAAGAAAGTCACCAATGCCGTTAAGAAAGTCGTTAAGGAAGGGGATCGTCCGGTTAAAAAGGCTGCTGGCGGTCTATCTGGTCTGGCTGGTTGTACTGGTGAAGCACAGATGGTGGAAGTACCACAAGCTGCCGGACAGATAGTTGACGTATCTGAGAAGGGGGTTTCCACTGAGGACGAAGCACCGACAGAAAGCGGACGCAAGAAATCTCGTGCTGGCGGTAAGACATCCTTGAGTGTAGCCCGTATCTCCGGTGGCGGTATCAACATTTAATCAGGAGGTTATCg                                                                                         <  1:313173/294‑1 (MQ=255)`  
                                                                                                                                                                                                                        `agaagGCCGTGAAGAAAGTCACCAAGTCCGTTAAGAAAGTCGTTAAGGAAGGGGCTCGTCCGGTTAAACAGGTTGCTGGCGGTCTAGCTGGTCTGGCTGGTGGTACTGGTGAAGCACAGATGGTGGAAGTACCACAAGCTGCCGCACAGATTGTTGACGTACCTGAGAAAGAGGTTTCCACTGAGGACGAAGCACAGACAGAAAGCGGACGCAAGAAAGCTCGTGCTGGCGGTAAGAAATCCTTGAGTGTAGCCCGTAGCTCCGGTGGCGGTATCAACATTTAATCAGGAGGTTATCGTgg                                                                                      <  1:17484/301‑1 (MQ=255)`  
                                                                                                                                                                                                                                  `aaggaaAGTCACCAAGTCCGTTAAGAAAGTCGTTAAGGAAGGGGCTCGTCCGGTTAAACAGGTTGCTGGCGGTCTAGCTGGTCTGGCTGGTGGTACTGGTGAAGCACAGATGGTGGAAGTACCACAAGCTGCCGCACAGATTGTTGACGTACCTGAGAAAGAGGTTTCCACTGAGGACGAAGCACAGACAGAAAGCGGACGCAAGAAAGCTCGTGCTGGCGGTAAGAAATCCTTGAGTGTAGCCCGTAGCTCCGGTGGCGGTATCAACATTTAATCAGGAGGTTATCGTGGAAGACTGCAt                                                                            <  1:507970/298‑1 (MQ=255)`  
                                                                                                                                                                                                                                    `agaaAGTCACCAAGTCCGTTAAGAAAGTCGTTAAGGAAGGGGCTCGTCCGGTTAAACAGGTTGCTGGCGGTCTAGCTGGTCTGGCTGGTGGTACTGGTGAAGCACAGATGGTGGAAGTACCACAAGCTGCCGCACAGATTGTTGACGTACCTGAGAAAGAGGTTTCCACTGAGGACGAAGCACAGACAGAAAGCGGACGCAAGAAAGCTCGTGCTGGCGGTAATAAATCCTTTAGTGTAGCCCGTAGCTCCGGTGGCGGTATCAACATTTAATCAGGAGGTTATCGTAGAAGACTGCATTg                                                                          <  1:663525/301‑1 (MQ=255)`  
                                                                                                                                                                                                                                       `tgtgtataagagacagtAAGAAAGTCGTTAAGGAAGGGGCTCGTCCGGTTAAACAGGTTGCTGGCGGTCTAGCTGGTCTGGCTGGTGGTACTGGTGAAGCACAGATGGTGGAAGTACCACAAGCTGCCGCACAGATTGTTGACGTACCTGAGAAAGAGGTTTCCACTGAGGACGAAGCACAGACAGAAAGCGGACGCAAGAAAGCTCGTGCTGGCGGTAAGAAATCCTTGAGTGTAGCCCGTAGCTCCGGTGGCGGTATCAACATTTAATCAGGAGGTTATCGTGGAAGACTGCATTGAAt                                                                       <  1:341229/285‑1 (MQ=255)`  
                                                                                                                                                                                                                                       `aaGTCACCAAGTCCGTTAAGAAAGTCGTTAAGGAAGGGGCTAGTCCGGTTAAACAGGTTGCTGGCGGTCTAGCTGGTCTGGCTGGTGGTACTGGTGAAGCACAGATGGTGGAAGTACCACAAGCTGCCGCACAGATTGTTGACGTACCTGAGAAAGAGGTTTCCACTGAGGACGAAGCACAGACAGAAAGCGGACGCAAGAAAGCTCGTGCTGGCGGTAAGAAATCCTTGAGTGTAGCCCGTAGCTCCGGTGGCGGTATCAACATTTAATCAGGAGGTTATCGTGGAAGACTGCATTGAAt                                                                       <  1:640872/301‑1 (MQ=255)`  
                                                                                                                                                                                                                                                     `gTTAAGAAAGTCGTTAAGGAAGGGGCTCGTCCGGTTAAACAGGTTGCTGGCGGTCTAGCTGGTCTGGCTGGTGGTACTGGTGAAGCACAGATGGTGGAAGTACCACAAGCTGCCGCCCAGATTGTTGACGTACCTGAGAAAGAGGTTTCCACTGCGGACGAAGCACAGACAGAAAGCGGACGCAAGAAAGCTCGTGCTGGCGGTAAGAAATCCTTGAGTGTAGCCCGTAGCTCCGGTGGCGGTATCAACATTTAATCAGGAGGTTATCGTGGAAGACTGCCTTGAATGGACCGCTGGTGTc                                                         >  1:473347/1‑301 (MQ=255)`  
                                                                                                                                                                                                                                                         `aGAAAGTCGTTAAGGAAGGGGCTCGTCCGGTTAAACAGGTTGCTGGCGGTCTAGCTGGTCTGGCTGGTGGTACTGGTGAAGCACAGATGGTGGAAGTACCACAAGCTGCCGCACAGATTGTTGACGTACCTGAGAAAGAGGTTTCCACTGAGGACGAAGCACAGACAGAAAGCGGACGCAAGAAAGCTCGTGCTGGCGGTAAGAAATCCTTGAGTGTAGCCCGTAGCTCCGGTGGCGGTATCAACATTTAATCAGGAGGTTATCGTGGAAGACTGCATTGAATGGACCGGAGGTGTCAAct                                                     <  1:441104/301‑1 (MQ=255)`  
                                                                                                                                                                                                                                                              `gTCGTTAAGGAAGGGGCTCGTCCGGTTAAACAGGTTGCTGGCGGTCTAGCTGGTCTGGCTGGTGGTACTGGTGAAGCACAGATGGTGGAAGTACCACAAGCTGCCGCACAGATTGTTGACGTACCTGAGAAAGAGGTTTCCACTGAGGACGAAGCACAGACAGAAAGCGGACGCAAGAAAGCTCGTGCTGGCGGTAAGAAATCCTTGAGTGTAGCCCGTAGCTCCGGTGGCGGTATCAACATTTAATCAGGAGGTTATCGTGGAAGACTGCATTGAATGGACCGGAGGTGTCAACTCTCAg                                                >  1:484085/1‑301 (MQ=255)`  
                                                                                                                                                                                                                                                               `tCGTTAAGGAAGGGGCTCGTCCGGTTAAACAGGTTGCTGGCGGTCTAGCTGGTCTGGCTGGTGGTACTGGTGAAGCACAGATGGTGGAAGTACCACAAGCTGCCGCACAGATTGTTGACGTACCTGAGAAAGAGGTTTCCACTGAGGACGAAGCACAGACAGAAAGCGGACGCAAGAAAGCTCGTGCTGGCGGTAAGAAATCCTTGAGTGTAGCCCGTAGCTCCGGTGGCGGTATCAACATTTAATCAGGAGGTTATCGTGGAAGACTGACTTGAATGGAGCGGAGGTGTCAACTCTaaag                                               >  1:88588/1‑299 (MQ=255)`  
                                                                                                                                                                                                                                                                `cGTTAAGGAAGGGGCTCGTCCGGTTACACAGGTTGCTGGCGGTCTAGCTGGTCTGGCTGGTGGTACTGGTGAAGCACAGATGGTGGAAGTACCACAAGCTGCCGCACAGATTGTTGACGTACCTGAGAAAGAGGTTTCCACTGAGGACGAAGCACAGACAGAAAGCGGACGCAAGAAAGCTCGTGCTAGCGGTAAGAAATCCTTGAGTGTAGCCCGTAGCTCCGGTGGCGGTATCAACATTTAATCAGGAGGTTATCGTGGAAGACTGCATTGAATGGACCGGAGGTGTCAACTCTAAggg                                              <  1:347343/301‑1 (MQ=255)`  
                                                                                                                                                                                                                                                                        `aaggGGCTCGACCGGTTAAACAGGTTGCTGGCGGTCTAGCTGGTCTGGCTGGTGGTACTGGTGAAGCACAGATGGTGGAAGTACCACAAGCTGCCGCACAGATTGTTGACGTACCTGAGAAAGAGGTTTCCACTGAGGACGAAGCACAGACAGAAAGCGGACGCAAGAAAGCTCGTGCTGGCGGTAAGAAATCCTTGAGTGTAGCCCGTAGCTCCGGTGGCGGTATCAACATTTAATCAGGAGGTTATCGTGGAAGACTGCATTGAATGGACCGGAGGTGTCAACTCTAAGGGTTATGGTc                                      <  1:229174/301‑1 (MQ=255)`  
                                                                                                                                                                                                                                                                             `gCTCGTCCGGTTAAACAGGTTGCTGGCGGTCTAGCTGGTCTGGCTGGTGGTACTGGTGAAGCACAGATGGTGGAAGTACCACAAGCTGCCGCACAGATTGTTGACGTACCTGAGAAAGAGGTTTCCACGGAGGACGAAGCACAGACAGAAAGCGGACGCAAGAAAGCTCGTGCTGGCTGTAAGAAATCCTTGAGTGTAGCCCGTAGCTCCGGTGGCGGTATCAACATTTAATCAGGAGGTTATCGTGGAAGACTGCATTGAATGGACCGGAGGTGTCAACTCTAAGGGTTATGGTCGTAAg                                 <  1:126679/301‑1 (MQ=255)`  
                                                                                                                                                                                                                                                                              `cTCGTCCGGTTAAACAGGTTGCTGGCGGTCTAGCTGGTCTGGCTGGTGGTACTGGTGAAGCACAGATGGTGGAAGTACCACAAGCTGCCGCTCAGATTGTTGACGTACCTGAGAAAGAGGTTTCCACTGAGGACGAAACACAGACAGAAAGCGGACGCAAGAAAGATCGTGCTGGCGGTAAGAAATCCTTGAGTGTAGCCCGTAGCTCCGGTGGCGGTATCAACATTTAATCAGGAGGTTATCGTGGAAGACTGCATTGAATGGACCGGAGGTGTCAACTCTAAGGGTTATGGTCGTAAgg                                >  1:289519/1‑300 (MQ=255)`  
                                                                                                                                                                                                                                                                              `cTCGTCCGGTTAAACAGGTTGCTGGCGGTCTAGCTGGTCTGGCTGGTGGTACTGGTGAAGCACAGATGGTGGAAGTACCACAAGCTGCCGCACAGATTGTTGACGTACCTGAGAAAGAGGTTTCCACTGAGGACGAAGCACAGACAGAAAGCGGACGCAAGAAAGCTCGTGCTGGCGGTAAGAAATCCTTGAGTGTAGCCCGTAGCTCCGGTGGCGGTATCAACATTTAATCAGGAGGTTATCGTGGAAGACTGCATTGAATGGACCGGAGGTGTCAACTCTAAGGGTTATGGTCGTAAGt                                <  1:29579/301‑1 (MQ=255)`  
                                                                                                                                                                                                                                                                                 `gTCCGGTTAACCAGGTTGCTGGCGGTCTAGCTGGTCTGGCTGGTGGTACTGGTGAAGCACAGATGGTGGAAGTACCTCAAGCTGCCGCACCGATTGTTTACGTACCTGAGAAAGAGGTTTCCACTGAGGACGAAGCACAGACAGCAAGCGGACGCAAGAAAGCTCGTGCTGGCGGTAAGAAATCCTTGAGTGTAGCCCGTAGCTCCGGTGGCGGTATCAACAATTAATCAGGAGGTTATCTTGGAAGACTGCATTGAATGGACCGGAGGTTTCAACTCTAAgggctgtctcttatccacat                             >  1:647182/1‑284 (MQ=255)`  
                                                                                                                                                                                                                                                                                    `cGGTTAAACAGGTTGCTGGCGGTCTAGCTGGTCTGGCTGGTGGTACTGGTGAAGCACAGATGGTGGAAGTACCACAAGCTGCCGCACAGATTGTTGACGTACCTGAGAAAGAGGTTTCCACTGAGGACGAAGCACAGACAGAAAGCGGACGCCAGAAAGCTCGTGCTGGCGGTAAGAAATCCTTGAGTGTAGCCCGTAGCTCCGGTGGCGGTATCAACATTTAATCAGGAGGATATCGTGGAAGACTGCATTGAATGGACCGGAGGTGTCAACTCTAAGGGTTAAGGTctgtctcttataa                          >  1:616403/1‑289 (MQ=255)`  
                                                                                                                                                                                                                                                                                      `gTTAAACAGGTTGCTGGCGGTCTAGCTGGTCTGGCTGGTGGTACTGGTGAAGCACAGATGGTGGAAGTACCACAAGCTGCCGCACAGATTGTTGACGTACCTGAGAAAGAGGTTTCCACTGAGGACGAAGCACAGACAGAAAGCGGACGCAAGAAAGCTCGTGCTGGCGGTAAGAAATCCTTGAGTGTAGCCCGTAGCTCCGGTGGCGGTATCAACATTTAATCAGGAGGTTATCGTGGAAGACTGCATTGAATGGACCGGAGGTGTCAACTCTCAGGGTTATGGTCGTAAGTGGGTTAAt                        >  1:276813/1‑301 (MQ=255)`  
                                                                                                                                                                                                                                                                                             `atgTTGCTGGCGGTCTAGCTGGTCTGGCTGGTGGTACTGGTGAAGCACAGATGGTGGAAGTACCACAAGCTGCCGCACAGATTGTTGACGTACCTGAGAAAGAGGTTTCCACTGAGGACGAAGCACAGACAGAAAGCGGACGCAAGAAAGCTCGTGCTGGCGGTAAGAAATCCTTGAGTGTAGCCCGTAGCTCCGGTGGCGGTATCAACATTTAATCAGGAGGTTATCGTGGAAGACTGCATTGAATGGACCGGAGGTGTCAACTCTAAGGGTTATGGTCGTAAGTGGGTTAATGGTAAAc                 <  1:353691/299‑1 (MQ=255)`  
                                                                                                                                                                                                                                                                                                       `cGGTCTAGCTGGTCTGGCTGGTGGTACTGGTGAAGCACAGATGGTGGAAGTACCACAAGCTGCCGCACAGATTGTTGACGTACCTGAGAAAGAGGTTTCCACTGAGGACGAAGCACAGACAGAAAGCGGACGCAAGAAAGCTCGTGCTGGCGGTAAGAAATCCTTGAGTGTAGCCCGTAGCTCCGGTGGCGGTATCAACATTTAATCAGGAGGTTATCGTGGAAGACTGCATTGAATGGACCGGAGGTGTCAACTCTAAGGGTTATGGTCGTAAGTGGGTTAATGGTAAACTTGTGACTcc       >  1:371067/1‑301 (MQ=255)`  
                                                                                                                                                                                                                                                                                                         `gtgtAGCGGGTCTGGCTGGGGGGAATGGTGAAGCACAGCTGGTGGAAGTACCACAAGCTGCCGAACAGATTGTTGACGTAACTGAGAAAGAGGTTTGCACTTAGGACGAAGCACAGACAGAAAGCGGACGCAAGAAAGCTCGTGATGGCGGTAAGAAATCCTTGAGTGTAGCCCGTAGCTCCGGTGGCGGTATCAACATTTAATCAGGAGGTTATCGTGGAAGACTGCATTGAATGGACCGGAGGTGTCAAATCTAAGGGTTATGGTCGTAAGTGGGTTAATGGTAAACTTGTGACTCcac     <  1:364968/298‑1 (MQ=255)`  
                                                                                                                                                                                                                                                                                                           `cTAGCTGGTCTGGCTGGTGGTACTGGTGAAGCACAGATGGTGGAAGTACCACAAGCTGCCGCACAGATTGTTGACGTACCTGAGAAAGAGGTTTCCACTGAGGACGAAGCACAGACAGAAAGCGGACGCAAGAAAGCTCGTGCTGGCGGTAAGAAATCCTTGAGTGTAGCCCGTAGCTCCGGTGGCGGTATCAACATTTAATCAGGAGGTTATCGTGGAAGACTGCATTGAATGGACCGGAGGTGTCAACTCTAAGGGTTATGGTCGTAAGTGGGTTAATggctgtctcttatacacatct   >  1:32364/1‑282 (MQ=255)`  
                                                                                                                                                                                                                                                                                                            `tAGCTGGTCTGGCTGGTGGTACTGGTGAAGCACAGATGGTGGAAGTACCACAAGCTGCCGCACAGATTGTTGACGTACCTGAGAAATAGGTTTCCACTGAGGACGCAGCACAGACAGAAAGCGGACGCAAGAAAGCTCGTGCTGGCGGTAAGAAATCCTTGAGTGTAGCCCTTAGCTCCGGTGGCGGTATCAACATTTAATCAGGCGGTTATCGTGGAAGACTGCATTTAATGGACCGGAGTTTTCAACTCTACGGGTTATGGTCGTACTTGGGTTAATGTTAAACTTGTGACTCCACATa  >  1:448355/1‑301 (MQ=255)`  
                                                                                                                                                                                                                                                                                                            `tAGCTGGTCTGGCTGGTGGTACTGGGGAAGCAAAGATGGTGGAAGTACCACAAGCTGCGGGACAGATTGTGGACGTACCTGAGAAAGAGGTTTCCACTGAGGACGAAGCATCGACAGAAAGCGCACGCAATAAATCTCTTGCTGGCGGTAAGAAATCCTTGAGTGTAGCCCGTAGCTCCGGTGGCGGTATCAACATTTAATCAGGAGGTTTTCGTGGAAGACTGCATTGAATGGACCGGAGGTGTCAACTCTAAGGGTTATGGTCGTAAGTGGGTTAATGGTAAACTTGTGACTCCACATa  <  1:424559/301‑1 (MQ=255)`  

                                                                                                                                                                                                                                                                                                            `|`                                                                                                                                                                                                                                                                                                              
`TAAGCTGTGACATTCACCACAAAGGAGACATTCTGGATGTAACGTCCGTTGTCATTAATCCTGAGGCAGACTCTAAGGGCTTACAGCGATTCCTAGCGAAACGCTTTAAGTACCTTGCGGAACTCCACGATTGCGATTGGGTGTCTCGTTGTAAGCATGAAGGCGAGACAATGCGTGTATACTTTAAGGAGGTATAAGTTATGGGTAAGAAAGTTAAGAAGGCCGTGAAGAAAGTCACCAAGTCCGTTAAGAAAGTCGTTAAGGAAGGGGCTCGTCCGGTTAAACAGGTTGCTGGCGGTCTAGCTGGTCTGGCTGGTGGTACTGGTGAAGCACAGATGGTGGAAGTACCACAAGCTGCCGCACAGATTGTTGACGTACCTGAGAAAGAGGTTTCCACTGAGGACGAAGCACAGACAGAAAGCGGACGCAAGAAAGCTCGTGCTGGCGGTAAGAAATCCTTGAGTGTAGCCCGTAGCTCCGGTGGCGGTATCAACATTTAATCAGGAGGTTATCGTGGAAGACTGCATTGAATGGACCGGAGGTGTCAACTCTAAGGGTTATGGTCGTAAGTGGGTTAATGGTAAACTTGTGACTCCACATA  >  NC_001604.1/19335‑19935`

  

Alignment Legend

Aligned base mismatch/match (shaded by quality score): `ATCG/ATCG` `< 3 ≤` `ATCG/ATCG` `< 10 ≤` `ATCG/ATCG` `< 20 ≤` `ATCG/ATCG` `< 34 ≤` `ATCG/ATCG` `< 38 ≤` `ATCG/ATCG`

Unaligned base: `atcg`    Masked matching base: `atcg`    Alignment gap: `‑`    Deleted base: `‑`

Reads not counted as support for junction

read\_name Not counted due to insufficient overlap past the breakpoint.

read\_name Not counted due to not crossing MOB target site duplication.
